# The multikinase inhibitor Sorafenib enhances glycolysis and synergizes with glycolysis blockade for cancer cell killing

**DOI:** 10.1038/srep09149

**Published:** 2015-03-17

**Authors:** Valentina Tesori, Anna Chiara Piscaglia, Daniela Samengo, Marta Barba, Camilla Bernardini, Roberto Scatena, Alessandro Pontoglio, Laura Castellini, Johannes N. Spelbrink, Giuseppe Maulucci, Maria Ausiliatrice Puglisi, Giovambattista Pani, Antonio Gasbarrini

**Affiliations:** 1 Institute of Internal Medicine and Gastroenterology, Catholic University of the Sacred Heart School of Medicine; 2 Institute of General Pathology, Laboratory of Cell Signaling, Catholic University of the Sacred Heart School of Medicine; 3 Institute of Human Anatomy and Cell Biology, Catholic University of the Sacred Heart School of Medicine; 4 Institute of Biochemistry and Clinical Biochemistry, Catholic University of the sacred Heart School of Medicine; 5 Department of Radiation Oncology, Center for Clinical Sciences Research, Stanford University School of Medicine, Stanford, CA 94305, USA; 6 Department of Pediatrics, Nijmegen Centre for Mitochondrial Disorders, Radboud University Medical Centre, Geert Grooteplein 10, P.O. Box 9101, 6500 HB Nijmegen, The Netherlands; Institute of Biomedical Technology & Tampere University Hospital, Pirkanmaa Hospital District, University of Tampere, FI-33014, Finland; 7 Institute of Physics, Catholic University of the Sacred Heart School of Medicine

## Abstract

Although the only effective drug against primary hepatocarcinoma, the multikinase inhibitor Sorafenib (SFB) usually fails to eradicate liver cancer. Since SFB targets mitochondria, cell metabolic reprogramming may underlie intrinsic tumor resistance. To characterize cancer cell metabolic response to SFB, we measured oxygen consumption, generation of reactive oxygen species (ROS) and ATP content in rat LCSC (Liver Cancer Stem Cells) -2 cells exposed to the drug. Genome wide analysis of gene expression was performed by Affymetrix technology. SFB cytotoxicity was evaluated by multiple assays in the presence or absence of metabolic inhibitors, or in cells genetically depleted of mitochondria. We found that low concentrations (2.5–5 μM) of SFB had a relatively modest effect on LCSC-2 or 293 T cell growth, but damaged mitochondria and increased intracellular ROS. Gene expression profiling of SFB-treated cells was consistent with a shift toward aerobic glycolysis and, accordingly, SFB cytotoxicity was dramatically increased by glucose withdrawal or the glycolytic inhibitor 2-DG. Under metabolic stress, activation of the AMP dependent Protein Kinase (AMPK), but not ROS blockade, protected cells from death. We conclude that mitochondrial damage and ROS drive cell killing by SFB, while glycolytic cell reprogramming may represent a resistance strategy potentially targetable by combination therapies.

Aerobic glycolysis (“Warburg effect”) represents one of the distinctive tracts (“hallmarks”) of the malignant phenotype^1^,^2^,^3^,^4^. Although energetically less efficient than respiration, fermentative metabolism is advantageous for cell growth due to the increased availability of anabolic intermediates and the reduced cell dependence on oxygen; moreover, by increasing intracellular reducing equivalents (NADPH and glutathione) and decreasing mitochondria-derived ROS, glycolysis protects malignant cells from oxidant-induced senescence and apoptosis^5^ and contributes to the survival of Cancer Stem Cell (CSC)^6^. Biochemical differences between cancerous and normal cells may help directing targeted therapies against malignant elements. For instance, tumor cells are often strongly dependent on glucose (“glucose-addicted”) and therefore exquisitely sensitive to the glycolytic inhibitor 2-deoxyglucose (2DG)^7^. Notably, the link between metabolism, oxidative stress and cancer may be particularly relevant to the liver^8^, that plays a pivotal role in the regulation of glucose homeostasis. Hence liver cancer cells, like the hepatocholangiocarcinoma cell line LCSC-2 we've recently derived from a novel model of carcinogenesis in rats^9^, appear ideally suited to investigate biochemical mechanisms and therapeutic implications of cancer cell metabolic reprogramming.

The multikinase inhibitor Sorafenib (SFB) (Nexavar, BAY 43-9006) currently represents the primary treatment option for advanced hepatocellular carcinoma^10^; SFB preferentially inhibits the cancer- associated V600E mutant of the serine-threonine kinase and Ras-effector BRAF, while the wild type enzyme is paradoxically activated by the drug in the presence of active Ras signaling^11^; SFB also targets, at concentrations in the high nanomolar range, a number of Receptor Tyrosine Kinases (RTK_S_) including, Platelet Derived Growth Factor – β (PDGFR-β), Vascular Endothelial Growth Factor-2 (VEGFR-2), and Vascular Endothelial Growth Factor-2 (VEGFR-3)^12^. However, additional mechanisms likely contributeto the elevated anticancer activity of this compound, and may have by extension a role in the frequent emergence of specific chemoresistance^13^.

Initial evidence point to mitochondrial damage and oxidative stress as additional, kinase-independent mechanisms underlying cell response to Sorafenib. In normal cardiomyocytes, for instance, SFB was reported to inhibit mitochondrial respiration and to decrease intracellular ATP levels^14^. Along similar lines, SFB has been shown to increase the production of mitochondrial ROS (mROS), decrease reduced Glutathione levels (GSH) and induce cell death in HepG2 human hepatoma cells^15^, and serum levels of advanced oxidation protein products in Sorafenib-treated HCC patients correlate with clinical effectiveness of the drug^16^. Additionally, in human pancreatic cell lines SFB elicits MEK/ERK independent apoptosis, through the downregulation of the mitochondrial antiapoptotic protein Mcl-1^17^.

Prompted by these evidence and by the emerging interest towards metabolism-targeted anticancer therapies, we sought to investigate the effect of Sorafenib on mitochondrial activity and oxidative metabolism in rat hepatocolangiocrcinoma LCSC-2 cells, in search for novel mechanisms of response and/or resistance of liver cancer cells to this increasingly used drug.

## Results

### Sorafenib increases intracellular ROS and inhibits respiration in LCSC-2 cells

**Figure 1:**
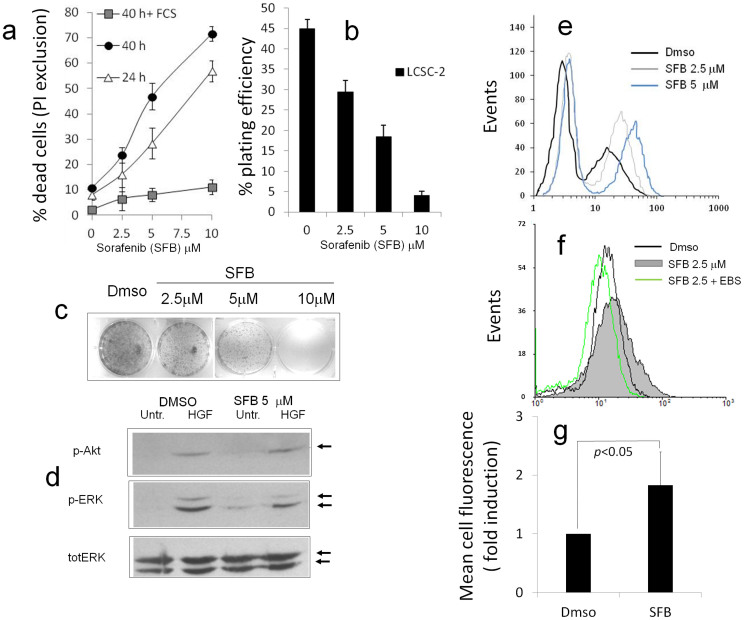
Growth inhibition and generation of ROS in LCSC-2 cells exposed to Sorafenib. (a), (b) and (c) Propidium Iodide exclusion assay (a) and colony formation assay (b and c) showing dose and time-dependent growth-inhibitory effect of SFB on LCSC-2 cells. FCS = fetal calf serum. In b, bars represent plating efficiency (n° colonies/n° of plated cells); c:representative picture of colonies stained with Giemsa. In a and b bars are mean ± SD of duplicate or triplicate samples. Panels representative of several independent experiments. (d). Western Blot analysis of Akt (Ser 473) and p44-42 MAP Kinase (ERK,Thr202/Tyr204) phosphorylation after 12 hours incubation with the indicated combinations of Hepatocyte Growth factor (HGF, 50 ng/ml) and SFB. Anti total ERK immune-staining confirms equal protein loading throughout the lanes. Relevant bands are indicated by arrows. (e). Representative flow cytometry plot revealing broad distribution of H2-DCFDA fluorescence in LCSC-2 cells and increased signal (oxidation) in response to 2.5 or 5 μM SFB. (f). Effect of antioxidants and GPX mimetic Ebselen on SFB-induced ROS. A shift of cell fluorescence profile to the left confirms effective ROS scavenging by the compound. Plots representative of several independent analyses. (g). Quantitation of ROS increase in LCSC-2 cells exposed to 2.5 μM SFB for 12 hours. Values are Mean ± SD of mean fluorescence ratios (SFB/Dmso) over n = 6 independent experiments. *p* calculated on raw fluorescence values by paired two-tailed t-test.

Sensitivity of tumor cell lines to RTKs inhibitors is highly variable, in part depending on the mutational status of RAS and RAF family members^18^. Exposure of rat hepatocolangiocarcinoma LCSC-2 cells, that lack B-RAF activating mutations, to SFB had a modest growth inhibitory effect as assessed by Propidium Iodide (PI) exclusion or colony formation assay (Fig. 1, a, b and c), especially in the presence of fetal bovine serum: in fact, unlike reported for higly sensitive cell lines^12^, 50% inhibition was attained in the low micromolar, rather than nanomolar range. Of note, in this range no obvious reduction of phosphorylated (active) ERK and AkT, was observed under cell stimulation with Hepatocyte growth Factor (HGF), suggesting a growth inhibitory mechanism distinct from RTK or ERK blockade (Fig. 1, d).

Consistent with previous reports^15^,^16^, flow cytometry analysis of cells loaded with the redox-sensitive dye H2-DCF-DA revealed a marked and dose dependent increase of ROS 12 hours after exposure to 2,5 or 5 μM SFB (Fig. 1 e and 1 g); LCSC-2 cells often appeared distributed in two distinct subpopulation peaks based on DCF-DA fluorescence intensity, both of which were shifted to the right (increased oxidation) upon exposure to the drug (Fig. 1e). Importantly, the pro-oxidant effect of SFB was cancelled by the cell permeant hydrogen peroxide scavenger and GPX mimetic Ebselen (EBS) (Fig. 1 f).

**Figure 2:**
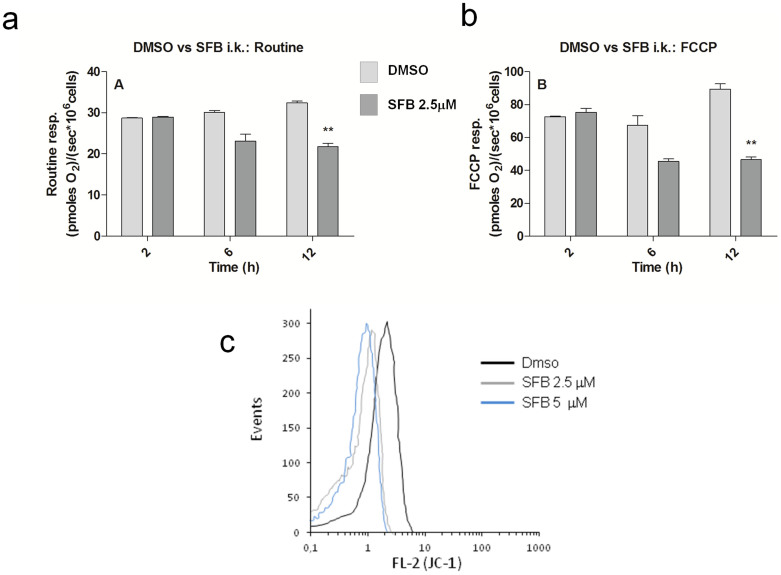
Sorafenib inhibits cellular respiration and depolarizes isolated mitochondria. (a) and (b). Respirometric Analysis of LCSC-2 cells exposed for 2, 6 and 12 hours to 2.5 μM SFB or Dmso as vehicle control. The two histograms illustrate the inhibitory effect of the drug on Routine Respiration (A), and uncoupled respiration (B) (see methods for experimental details). Asterisks denote statistical significance (two-tailed Student t-test, compared to untreated control). (c). Flow cytometry analysis of mitochondrial staining with the potentiometric dye JC-1. Decreased red (FL-2) fluorescence of mitochondria exposed to SFB indicates loss of trans-membrane potential (ψ). Plot representative of two independent experiments.

Since mitochondria represent the main sources of ROS in normal and in tumor cells, we reasoned that ROS increase may reflect an effect of SFB on these organelles. Accordingly, baseline oxygen consumption (routine respiration) measured by High Resolution Respirometry was significantly reduced after 12 hours incubation with SFB, compared to untreated controls (Fig. 2 a), and a similar difference was observed under maximum electron flow, as elicited by the mitochondrial protonophore Carbonyl cyanide p-(tri-fluoromethoxy) phenyl-hydrazone (FCCP), (Fig. 2 b).

In order to address whether SFB acts directly on mitochondria, we isolated the organelles from rat liver and assessed their transmembrane potential in the presence of the drug, using the cationic fluorescent dye JC-1 followed by flow cytometry. As shown in Fig. 2, c, SFB was able to depolarize mitochondria *in vitro*, as revealed by an evident reduction of JC-1 fluorescence in the FL2 (red) channel. Taken together, these findings confirm that in LCSC-2 cells, as in other cell models, SFB interferes with mitochondrial function, and suggest that this effect is due, at least in part, to a direct interaction of the drug with the organelle.

### Sorafenib affects energy metabolism of LCSC2 cells

**Figure 3:**
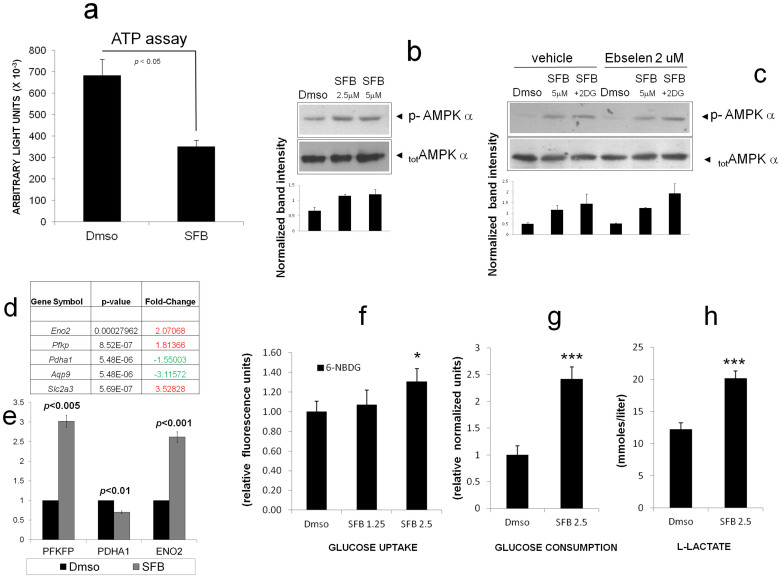
Sorafenib modulates energy metabolism. (a). ATP measurement in LCSC-2 cells treated with SFB. Values are in light units normalized for protein content. Bars are Mean ± SD of duplicate sample. Statistics are by two-tailed t-test. Histogram representative of two independent experiments. (b). Immunodetection of phospho-(Thr 172) AMPK phosphorylation under the indicated stimuli; anti AMPK (total) immunoblotting was used as loading control. (c). Anti p-AMPK immunoblot showing no inhibitory effect of Ebselen on AMPK phosphorylation by SFB or SFB + 2DG. Total AMPK was used as loading control. Relevant bands are indicated by arrows. Picture representative of at least two independent experiments. Black colums are Mean ± SD of densitometric values from 2–3 independent experiments, normalized for average band indesity. (d). Glycolysis-related genes modulated by Sorafenib in LCSC-2 cells. Fold change refers to the untreated (Dmso) sample. Upregulated genes are highlighted in red, downregulated genes in green. (e). Real Time PCR validation of three selected genes from panel C, a. Fold change (SFB/Dmso) for each gene was calculated by the ΔCt method (see Supplementary Methods); bars are mean ± SD of three independent reactions. Statistics are by two-tailed t-test. (f), (g) and (h). Bar graphs displaying enhanced 6NBDG uptake and increased glucose metabolism in LCSC-2 cells after 48 hours exposure to SFB. Bars are Mean SD of 2–3 independent samples. * p < 0.05 (ANOVA); *** p < 0.0001 (*t*-test).

To further characterize the impact of SFB on cellular energy metabolism, we first measured intracellular ATP after twelve hours cell exposure to the drug. ATP levels were markedly reduced (>50%) in SFB-treated cells compared to untreated controls (Fig. 3 a), indicating that LCSC-2 cells actively utilize mitochondria for their energy supply. In keeping with the above finding, SFB elicited the phosphorylation on Threonine 172 of the AMP activated protein kinase (AMPK), an energy sensor that detects changes in the intracellular AMP/ATP and triggers metabolic cell adaptations aimed at restoring ATP levels at expense of anabolic processes (Fig. 3 b). SFB phosphorylation of AMPK was potentiated by the glycolytic inhibitor 2DG (Fig. 3 c and Supplementary Figure S5), and was not inhibited by Ebselen (Fig. 3 c), although AMPK activation may in some contexts respond to ROS^19^.

To gain insight in the global metabolic cell response to Sorafenib, we performed microarray analysis of gene expression. A total of 322 differentially-expressed genes with ≥1,5 fold change were identified in LCSC-2 exposed to the drug. Among these, 174 genes resulted up-regulated and 148 genes were down-regulated in LCSC-2 treated with SFB compared to untreated controls (*data accessible at http://www.ncbi.nlm.nih.gov/geo/ under the ID number GSE43053*). Of note, monosaccaride metabolism (p = 0.00462) and cell proliferation (p = 0.00631) were among the highest ranking biological processes enriched for differently expressed genes (Supplementary Fig. S1 on line).

We focused on genes involved in glucose metabolism. Interestingly, the expression of three genes directly involved in glycolysis [the solute carrier family 2 (*slc2a3*), Enolase 2 (*eno2*), and the platelet phosphofructokinase (*pfkp*)], was significantly induced by SFB (Fig. 3 d); conversely, SFB decreased the expression of acquaporin 9 (*aqp9*), member of a family of water-selective membrane channels, and of the mitochondrial enzyme pyruvate dehydrogenase (lipoamide) alpha 1 (*pdha1*), which catalyzes the irreversible conversion of pyruvate to acetyl-CoA, thus linking aerobic glycolysis with the tricarboxylic acid (TCA) cycle in mitochondria. Differences in the expression levels of selected genes were further validated by quantitative real time PCR (qPCR) (Fig. 3 e). Moreover, consistent with metabolic shift towards glycolysis in response to SFB, suggested by the above transcriptional changes, we observed enhanced uptake of the fluorescent glucose analog 6NDBG, and increased glucose consumption and L-lactate release by LCSC-2 cells exposed to the drug (Fig. 3 f, g and h).

### Sorafenib toxicity is increased by the glycolytic inhibitor 2-deoxy-glucose

The above findings propted us to test whether the effects of SFB could be enhanced by a glycolysis inhibitor, such as the non-metabolizable glucose analogue 2-deoxy-glucose (2DG).

**Figure 4:**
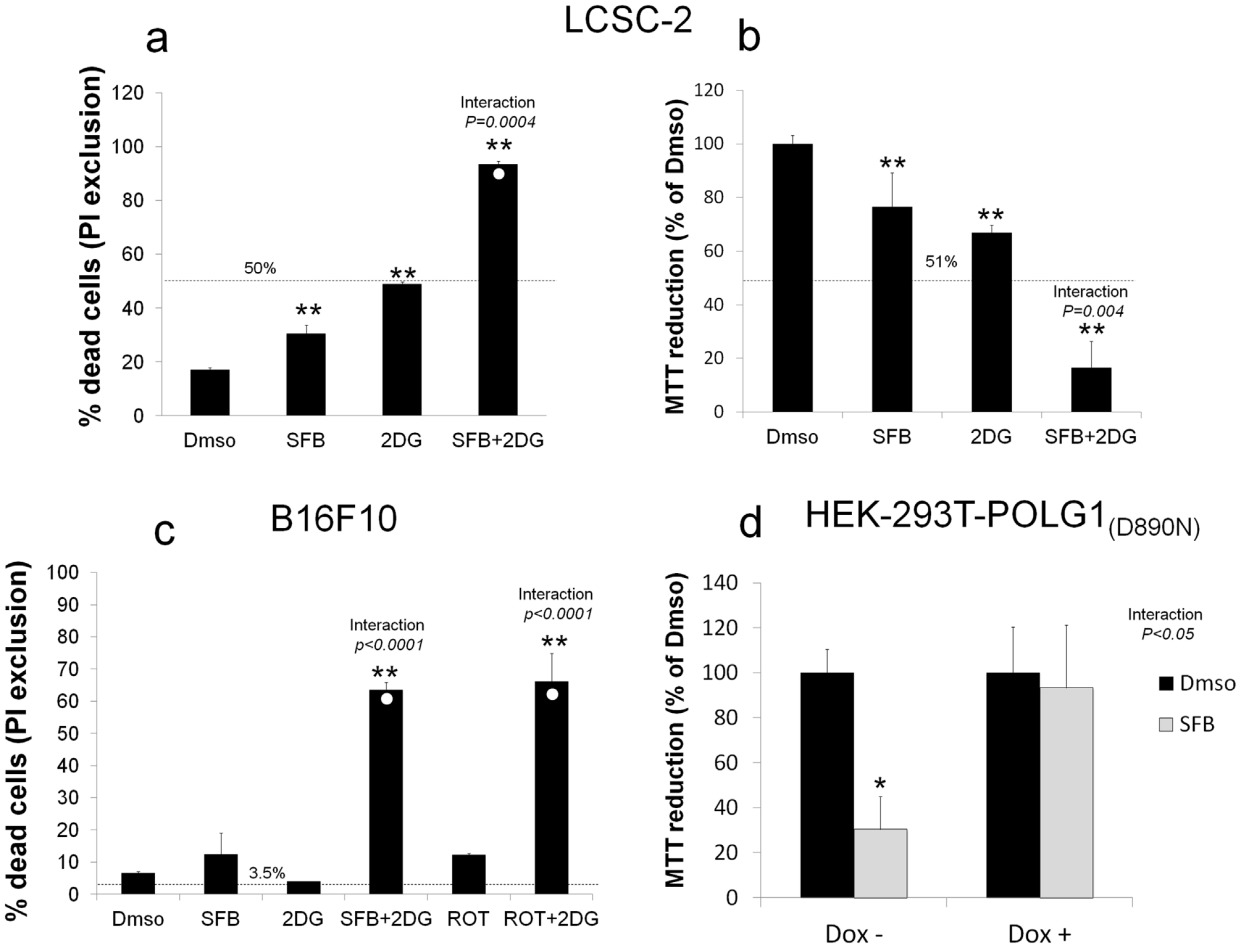
Sorafenib toxicity is increased by the glycolytic inhibitor 2-deoxy-glucose. (a). Cytotoxic effect of SFB (5 μM) and 2DG (20 mM), applied as single agents or in combination, in LCSC-2 cells. Percentage of dead (Propidium Iodide-permeant) cells was determined by flow cytometry after 24 hours exposure to the drugs. The dashed line indicates the expected value for simple additivity, cleared of background cell death (Dmso), calculated by the Bliss Independence Model (see Supplementary Methods). A white circle in the SFB + 2DG bar indicates the amount of cell death entirely attributable to the drug combination, cleared of the background. ** indicates p < 0.01 compared to vehicle; *interaction* denotes a significant “cell (row × column) effect” in the two-way ANOVA test. Histogram representative of several independent experiments (b). MTT Assay under the same conditions as in a. Bars are mean ± SD of three independent experiments, each in duplicate. Line and statistics are as in panel a. (c). Evaluation of SFB toxicity and SFB + 2DG interaction in B16 mouse melanoma cells. Cells were analysed for PI exclusion as in a, after 24 hours exposure to the drugs. Rotenone (Rot, 5 μM), mimics the effect of SFB (2.5 μM), and synergizes with 2DG. Bars are mean ± SD of duplicate samples. * = p < 0.05 and ** = p < 0.01 compared to Dmso. Statistics as in panel a. Panel representative of two independent experiments. (d) MTT assay showing lack of response to SFB (5 μM) of HEK293 –POLG1 (D890N) cells grown in doxycycline 50 ng/ml for 10 days to deplete mtDNA (Dox+); Dox- are non-induced cells that retain mtDNA. Picture representative of two independent experiments. Statistics as in panel a.

In toxicity assays (PI exclusion and MTT), 2DG dramatically increased cell killing by SFB both in LCSC-2 cells (Fig. 4 a and b) and in the highly malignant murine melanoma cell line B16F10 (Fig. 4 c); a similar effect was elicited by glucose withdrawal from the culture medium (Supplementary Fig. S2 on line). Of note, the MEK inhibitor PD98059 had marginal effects on LCSC-2 cells, that was not enhanced by glucose withdrawal (Supplementary Fig S2 on line). Instead, in B16F10 cells, the effect of Sorafenib was mimicked by the mitochondrial Complex I inhibitor Rotenone^20^ (Fig. 4 c); moreover, depletion of mitochondrial DNA in a HEK293 cell line variant expressing a doxycycline inducible dominant negative mutant of the mitochondrial DNA polymerase gamma 1 (POLG1 D890N)^21^ nearly abrogated sensitivity to SFB (fig. 4d), further pointing to a mitochondrial action of the drug. The synergistic effect of SFB and 2DG for cell killing was also confirmed in a number of additional human and murine cancer cell lines, including Ras-transformed Mouse Embryonic Fibroblasts (MEF) lacking p53 (Supplementary Fig. S3 on line).

### AMPK links metabolic damage by SFB to the mTOR/autophagy cascade

**Figure 5:**
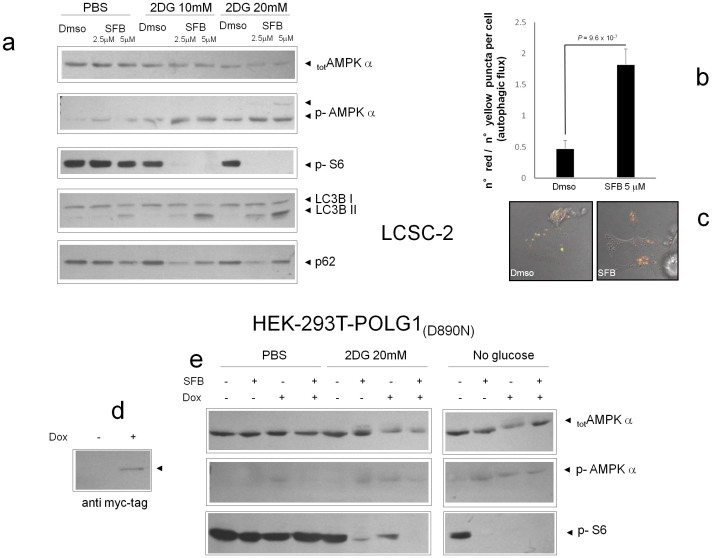
AMPK links metabolic damage by SFB to the mTOR/autophagy cascade. (a). Western blot analysis of protein lysates from LCSC-2 stimulated for 4–6 hours with the indicated combinations of SFB and 2DG. Phosphorylation of AMP (Ser 172) and of the mTOR effector S6 (Ser 235/236) were monitored by phospho-specific antibodies. Total AMPK is also displayed. In the lowest two panels, appearance of a fast migrating LC3B band (LC3B II) and decreased anti-p62 signal in SFB and SFB + 2DG treated samples denote enhanced autophagy. Picture representative of three independent experiments with comparable results. (b). Confocal analysis of LCSC-2 cells transiently transfected with a tandem red-green flurescent tagged LC3 and exposed to SFB for 6 hours. Red puncta (autolysosomes) and yellow puncta (autophagosomes) were counted and the ratio calculated for each cell. a. Bars are mean ± SD of n = 51 (Dmso) and n = 32 (SFB); statistics by t-test. (c). representative fluorescent microphotographs displaying enhanced autophagic flux (prevalence of red over yellow dots) in the sample exposed to SFB. (d). Expression of myc-tagged POLG1 (D890N) in HEK293 cells ten days after cultivation in the presence of 50 ng/ml doxycyclin. (e). Induced (Dox+) and non induced (Dox−) HEK-293 POLG1 (D890N) cells were stimulated for 4 hours as indicated (SFB + = 5 μM) and phosphorylation of AMPK and S6 evaluated by immunoblotting as in a. Total AMPK is also depicted. In the latter panel, appearance of a faint slow migrating band above the main one confirms AMPK hyperphosphorylation. SFB has little effect on AMPK in this highly glycolytic cell line, unless glycolysis is inhibited (2DG and No Glucose). Mitochondria depletion cancels SFB stimulatory effect on AMPK.

To further characterize the biochemical events elicited by SFB and its combination with 2DG, we interrogated nutrient signalling events along the AMPK kinase pathway. In LCSC2 (Fig. 5 a and Supplementary Fig. S5) and B16F10 cells (Supplementary Fig. S4) SFB and 2DG combinatorially and dose-dependently increased the phosphorylation of AMPK on Ser 172, consistent with profound cell de-energization. Even more dramatically the combination inhibited the phosphorylation of the ribosomal S6 protein, a downstream effector of the mTOR (mammalian Target of Rapamycin)/S6 Kinase cascade that is negatively regulated by AMPK under nutrient deprivation^22^. Moreover, Similar studies performed on HEK293-POLG1 (D890N) cells showed that AMPK phosphorylation by SFB is favoured under glycolysis blockade by either 2DG or glucose deprivation, and that it requires active mitochondria, as indicated by the lack of response to the drug (or the presence of an opposite, inhibitory action) in mtDNA-depleted cells (Fig. 5 d).

Noticeably, as an additional output of AMPK/mTOR signalling modulation in LCSC2 cells, SFB and SFB + 2DG triggered cell autophagy, a self-eating process aimed at cell survival under starvation^23^; this was revealed, biochemically, by increased LC3B electrophoretic mobility (due to covalent conjugation of this phagosome-associated protein with phosphatidyl-ethanolamine), and degradation of the autophagy substrate p62 (Fig. 5 a and Supplementary Fig. S5), and, functionally, by an accelerated autophagic flux (i.e. autophagosome-autolysosome transition), monitored by confocal analysis of LCSC-2 cells transiently transfected with a mRFP-GFP tandem fluorescent-tagged LC3^24^ (Fig. 5 b and 5 c).

### LCSC-2 cell killing by SFB involves ROS and is counteracted by AMPK

**Figure 6:**
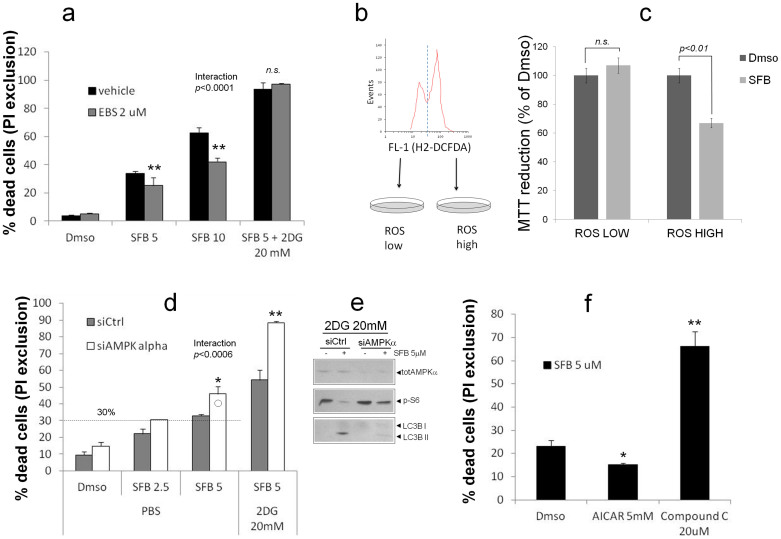
LCSC-2 cell killing by SFB involves ROS and is counteracted by AMPK. (a) PI exclusion assay depicting the effect of Ebselen (2 μM, gray bars) on LCSC-2 cell growth inhibition by SFB and SFB + 2DG. Bars are mean ± SD of duplicate or triplicate samples. Statistics are by two-way ANOVA. ** p < 0.01 compared to vehicle. Significance for the interaction SFBxEBS is also indicated. Representative of two independent experiments. (b). Identification and sorting of LCSC-2 cell populations with low and high content of ROS, based on H2-DCFDA fluorescence distribution on green fluorescence FL-1 histogram. (c). growth inhibition evaluated by MTT assay. Values are percentages of Dmso/PBS. Bars are mean ± SD of duplicate samples. Statistics of relevant comparisons by two-way ANOVA are indicated. Significance for the interaction effect (ROS x SFB) is indicated. Picture representative of two independent experiments. (d). Knock-down of AMPKα by siRNA sensitizes LCSC-2 cells to SFB. (e). PI exclusion assay on mock (siCtrl) and AMPK-silenced (siAMPKα) LCSC-2 cells exposed to SFB alone or in combination with 2DG for 24 hours. Significant differences (AMPK versus Ctrl) are indicated by asterisks. * p < 0.05; **p < 0.01 by two-way ANOVA. Bars are mean ± SD of duplicate samples. Picture representative of two independent experiments. (e). western blot analysis confirming AMPK downregulation and impaired pS6 inhibition/LC3B lipidation in siAMPK-treated cells exposed to SFB. Representative of two independent experiments. (f). PI exclusion assay illustrating LCSC-2 sensitivity to 5 μM SFB upon pharmacological modulation of AMPKα; inhibitors were added to cells 2 hours prior to SFB, and viability was assessed 22 hour later. * p < 0.05 ** p < 0.01 by one-way ANOVA. Values are mean ± SD of duplicate samples. Picture representative of two independent experiments.

In order to clarify the mechanistic role of ROS and phospho-AMPK, both elicited by SFB, in the cytotoxic activity of the drug, we first evaluated killing efficiency in LCSC2 cells pre-treated with the ROS scavenger Ebselen. The antioxidant significantly reduced cell death in the presence of 5 and 10 μM SFB (p < 0.01) (Fig. 6 a and Supplementary Fig. 6) confirming SFB-induced oxidative damage as a necessary contributor to the drug cytotoxic action. Accordingly, when LCSC-2 with high and low baseline levels of ROS (as revealed by the fluorescent dye DCF-DA) were separated by fluorescence-activated cell sorting, the two population displayed different sensitivity (sensitive the former, resistant the latter) to the drug (Fig. 6 b and c). Interestingly, however, Ebselen had no effect on massive cell killing by the SFB + 2DG combination (Fig. 6A and Supplementary Fig. 6), implying a ROS independent toxic mechanisms (energy depletion?) for the latter synergistic effect. On the other hand, AMPK depletion by siRNA technology (Fig. 6 d and e) potentiated cell killing by 5 μM SFB both alone and in combination with 2DG (p < 0.05 and p < 0.01, respectively), in parallel with impaired inhibition of mTOR/S6K signaling (Fig. 6 e). A cell protective role for AMPK in response to SFB was further corroborated by pharmacological modulation of the kinase with the cell permeant activator 5-Aminoimidazole-4-carboxamide 1-β-D-ribofuranoside (AICAR, 5 mM, p < 0.05), and the specific inhibitor Compound C (20 μM, p < 0.01). Thus, AMPK activation likely contributes to resistance to Sorafenib at least in LCSC-2 cells.

## Discussion

Cancer cell metabolism relies on a delicate balance between the glycolytic pathway, considerably amplified to provide for the needs of a rapid and invasive growth, and oxidative phosphorylation, which remains active, to contribute to the tumor energy needs^1^,^25^. Interference with this unique metabolic setting may open new possibilities for targeted anticancer therapy.

We have here investigated the metabolic effects of Sorafenib, a multikinase inhibitor, reported to preferentially target mutant (V600E) BRAF and a number of cancer-relevant tyrosine kinase receptors^12^. These effects, that include inhibition of mitochondrial respiration, reduced ATP production, and elevation of intracellular ROS, are in many cell lines, including the rat hepatocolangiocarcinoma cell line LCSC-2, largely insufficient to drive cell death; we here suggest that such ineffectiveness is due, to an efficient cell reprogramming towards aerobic glycolysis. The dramatic increase of SFB cytotoxicity by glucose withdrawal or the glycolytic inhibitor 2DG clearly supports this view, opening to the possibility of employing SFB in novel metabolism-based combination therapies. Importantly, SFB potentiation by glycolysis blockade was not limited to liver-derived cells, but also extended to melanoma cells and other cell types, and is likely to be independent from the tumor suppressor p53 (Supplementary Fig. S3).

Noteworthy, signaling studies (Fig. 1 d) indicate that metabolic actions of Sorafenib are independent from inhibition of RTK signaling, suggesting instead a direct interaction of the compound with mitochondria. Since mitochondria represent a novel promising substrate for targeted drugs^26^, molecular interactions of SFB with this organelle certainly deserve to be further investigated.

In keeping with the idea that SFB metabolically reprograms target cells, microarray data are consistent with a cell shift towards aerobic glycolysis, with a significant up-regulation of genes involved in the glycolytic pathway, such as *scl2a3* (also known as GLUT-3, a glucose transporter^27^), *pfkp* (Phosphofructokinase, platelet isoform^28^) and *eno2* (Enolase 2, a part of the phosphopyruvate hydratase complex^29^) (Supplementary Fig. S1). Importantly, all these three genes have been previously linked to human malignancy^30^,^31^,^32^. Conversely, among SFB-downregulated genes related to metabolism, the mitochondrial enzyme pyruvate dehydrogenase (lipoamide) alpha 1 (*pdha1*) catalyzes the irreversible conversion of pyruvate to acetyl-CoA, linking the glycolysis with the tricarboxylic acid (TCA) cycle and respiration^33^. Thus, overall, this gene signature points to a glycolytic adaptive response to mitochondrial failure, as it is also consistent with evidence of enhanced glucose consumption and lactate production in SFB-treated LCSC-2 cells (Fig. 3D).

Glycolysis provides, beside ATP and anabolic intermediates, also reducing equivalents (through the Pentose Phosphate Pathway, PPP) that maintain the GSH/GSSG redox buffer, and increased glycolysis may counteract SFB action, in part, by improving the cell antioxidant capacity^34^. In support of this view, LCSC-2 cells with constitutively low levels of ROS are intrinsically resistant, unlike cells with high ROS, to the drug action (Fig. 6 b and c). Interestingly, a similar Low-ROS profile identifies putative stem cells from normal and tumor breast cancer cell populations^6^, suggesting the possibility that resistance to SFB and a low oxidant content characterizes a subset LCSC-2 of cells with the properties of Cancer Stem Cells. By extension, resistance of CSC to SFB may explain the limited curative capacity of this drug^10^.With this respect it is noteworthy that glycolysis blockade overcomes SFB resistance in fashion that is ROS-independent (Fig. 6 a and Supplementary Fig. 6) and therefore circumvents, unlike SFB alone, potential antioxidants-based survival strategies implemented by cancer (stem) cells.

LCSC-2 resistance to Sorafenib also involves AMP kinase. Dose dependent induction of AMPK phosphorylation by SFB is most likely a consequence of cell de-energization, and appears to be independent of ROS (fig. 3 c) but requires active mitochondria, as shown by lack of response in HEK-293 cells grown in high glucose or devoid of mitochondrial DNA by a POLG1 dominant negative mutant (fig. 5 e and Supplementary Fig. S5). Moreover, a protective role for the kinase against SFB-induced cell death is strongly suggested by experiments of genetic and pharmacologic inhibition of the enzyme (fig. 6 d–f) and by correlative evidence of constitutive AMPK phosphorylation and resistance to SFB in HEK-293T POLG1 D890N cells (figs. 4 d, 5e and Supplemntary Fig. S5). Multiple protective mechanisms, including downregulation of mTOR signaling with reduced ATP consumption^35^, induction of autophagy (fig. 5 b and c), and genetic reprogramming (Supplementary Fig. S1) may account for this effect. While this aspect needs to be further clarified, the above observations are translationally relevant to the possibility of targeting AMPK in combination with SFB to increase cell response to the drug. Moreover, similar to other mitochondria-targeting drugs like Biguanides, tumor sensitivity to SFB may be predicted by loss of function of the tumor suppressor and AMPK activating kinase LKB1^36^, and boosted by limited glucose availability in the tumor mass^37^. Of note, while this manuscript was in preparation, it was reported that SFB antitumor action is mediated by AMPK/mTOR in breast cancer cells^38^, indicating that roles of AMPK downstream of this drug may be tumor- and context- dependent.

In conclusion, we have here provided novel evidence for important metabolic effects of the multikinase inhibitor Sorafenib on liver cancer cells. Our observations, although with the intrinsic limitations of studies in vitro, indicate in mitochondria an important target of the drug's action, involve ROS in SFB-dependent cytotoxicity, and identify multiple (AMPK, metabolic reprogramming, antioxidant capacity) potential mechanisms of drug resistance, to be circumvented by novel and more effective combination therapies.

## Methods

### Reagents and antibodies

Most of the Chemicals were obtained from Sigma-Aldrich (Milan, Italy). Sorafenib (Nexavar, BAY 43-9006) was kindly provided by Bayer Pharmaceuticals. Antibodies and siRNA are listed in SI.

### Plasmids

ptfLC3 (mRFP-GFP tandem fluorescent-tagged LC3) was a gift from Tamotsu Yoshimori (Addgene plasmid # 21074).

### Cell lines and transfections

The rat hepatocholangiocarcinoma cell line LCSC-2 was kindly provided by Dr. Thomas D. Shupe and Dr. Bryon E. Petersen (Dept. of Pathology, University of Florida, Gainesville, FL). Cells were maintained in Dulbecco's modified Eagle medium (DMEM 4.5 g/L d-glucose)/Ham's F12 medium 50:50, supplemented with 8% FBS and 1% antibiotics (*LCSC-medium*). Flp-In™ T-REx™ HEK-293 cells stably expressing the D890N dominant negative mutant of the mitochondrial Polymerase gamma (POLG1) under a Tetracycline-inducible promoter have been previously described^21^. Cell were routinely maintained in standard DMEM (4.5 g/L d-glucose). Induction of the recombinant POLG1-myc protein was readily detectable after 24 hours treatment with 50 ng/ml Doxycycline (Sigma), and was maintained for 10 days in order to severely deplete mitochondrial DNA. Additional cell lines are described in SI.

Transfection of cDNA and siRNA into LCSC-2 cells was performed with Lipofectamine® 2000 (Life Technologies) and HiPerFect (QIAGEN), respectively, according to the manufacturer's recommendations.

### Cytotoxicity assays

For MTT assay, cells were seeded in 24-well plates (2 × 10^5^/well) or 96 well plates (5 × 10^4^/well) and challenged with SFB other stimuli in serum-free medium.

After 24 hours of incubation MTT solution (1:10 dilution of the 5 mg/ml stock) was added to cells and incubated at 37°C for three additional hours. The MTT-containing medium was then removed, and the converted dye solubilized with 500 μl (24 well plate) or 100 μl (96 well plate) of acidic isopropanol (0.04 M HCl in absolute isopropanol or Isopropanol/Dmso 1:1 v/v. Absorbance was read at 570 nm.

For Propidium Iodide exclusion assay, medium containing floating cells was removed and attached cells were trypsinized and pooled with the floating ones in flow cytometry test tubes. Few seconds after addition of Propidium Iodide (1 μg/ml final concentration) cells were analysed for red fluorescence by flow cytometry (Exc. 488 nm, red fluorescence channel FL-3). Percentage of PI positive (dead) cells was determined, after exclusion of cell debris.

## Colony formation assay

For the determination of the colony formation capability, cells were seeded in 6-well plates (500/well) in complete culture medium. After two weeks cells were fixed with 3,7% Formalin for five hours and stained with 0.02% Giemsa.

### Intracellular ATP measurement

For intracellular ATP detection, cells were seeded in 12 well plates (4 × 10^5^/well/ml) in presence or in absence of relevant stimuli. After 12 h cells were processed and ATP was detected by using Promega kit, ENLITEN® ATP Assay System Bioluminescence Detection, according to the provider's recommendations^39^.

### High resolution respirometry on intact cells

A suspension of LCSC-2 in DMEM/F12, processed respectively with DMSO (ctrl) and Sorafenib 2.5 μM, was added to each Oxygraph Chamber (Chamber A and Chamber B), at a density of 0.5 × 10^6^ cells/ml.

The experiments began with the measurement of the Routine respiration (i.e. the respiration of drug-treated cells resuspended in the medium without the addiction of substrates), followed for about 10 minutes (STATE R). Then, 2 μl of 4 mg/ml Oligomycin, an ATP synthase blocker, were added in each chamber (STATE L) and respiration was recorded for 6 minutes. Subsequently, 5 μg/ml of FCCP (a protonophore - H^+^ ionophore - and uncoupler of oxidative phosphorylation) were added (STATE E) and respiration was recorded for 3 minutes; higher FCCP concentrations or wider/broader time intervals exert an inhibitory effect on cellular respiration depending on mitochondria.

All inhibitors and uncouplers used in the protocol are able to cross the cell membrane and do not require a prior cells permeabilization^40^.

### Confocal analysis of autophagic flux in LCSC-2 cells

Quantitative assessment of autophagosome to-autolysosome maturation dynamic the was performed according to^24^ using a fluorescent tagged LC3 probe.

### Isolation of mitochondria from rat liver

Mitochondria were isolated from the liver of an adult female Wistar rat, using the kit MITOISO1 from Sigma. Determination of organelle transmembrane potential with JC-1 was performed according to the kit instructions, as described elsewhere^41^.

## Author Contributions

G.P. and A.G. conceived and supervised the study; G.P. and V.T. carried out the experiments and analyzed data; D.S. participated in experimental procedures; G.M. performed confocal analysis of autophagic flux; A.C.P. and G.M. analyzed data; M.B., L.C. and C.B. carried out the Microarray Analysis; R.S. and A.P. performed the oxymetry experiments, J.N.S. contributed fundamental reagents and M.A.P. contributed to data analysis. G.P., V.T. and A.C.P. were involved in writing the paper. All the authors had final approval of the submitted version.

## Supplementary Material

Supplementary Information Supplementary Information
