# Supplementary material for: The multikinase inhibitor Sorafenib enhances glycolysis and synergizes with glycolysis blockade for cancer cell killing
Source: Sci Rep. 2015 Mar 17;5:9149. doi: 10.1038/srep09149 (PMC4361992; doi:10.1038/srep09149)

Full title:

The multikinase inhibitor Sorafenib enhances glycolysis and synergizes with glycolysis blockade for cancer cell killing.

Running title: Sorafenib induces cancer glycolytic switch

Authors and Affiliations.

Valentina Tesori[1], Anna Chiara Piscaglia[1], Daniela Samengo[2], Marta Barba[3], Camilla Bernardini[3], Roberto Scatena[4], Alessandro Pontoglio[4], Laura Castellini[5], Johannes N. Spelbrink[6], Giuseppe Maulucci[7], Maria Ausiliatrice Puglisi[1], Giovambattista Pani [2 †] and Antonio Gasbarrini [1]

[1] Institute of Internal Medicine and Gastroenterology, Catholic University of the Sacred Heart School of Medicine
[2] Institute of General Pathology, Laboratory of Cell Signaling, Catholic University of the Sacred Heart School of Medicine
[3] Institute of Human Anatomy and Cell Biology, Catholic University of the Sacred Heart School of Medicine
[4] Institute of Biochemistry and Clinical Biochemistry, Catholic University of the sacred Heart School of Medicine
[5] Department of Radiation Oncology, Center for Clinical Sciences Research, Stanford University School of Medicine, Stanford, CA 94305, USA.
[6] Department of Pediatrics, Nijmegen Centre for Mitochondrial Disorders, Radboud University Medical Centre, Geert Grooteplein 10, P.O. Box 9101, 6500 HB Nijmegen, The Netherlands; Institute of Biomedical Technology & Tampere University Hospital, Pirkanmaa Hospital District, University of Tampere, FI-33014, Finland.
[7]Institute of Physics, Catholic University of the Sacred Heart School of Medicine

[†] Corresponding author: Giovambattista Pani, MD, PhD, Largo F. Vito 1, 00168 Rome Italy, Ph 39 06 301254914; email: gpani@rm.unicatt.it

Supplementary Information.

- Supplementary Methods
- Supplementary References
- Supplementary Figure Legends
- 6 Supplementary Figures (S1 through S6).

Supplementary Methods.

Animals. Wistar rats were originally obtained from Charles River Italia (Calco, Lecco, Italy) and bred in the Animal Facility of Università Cattolica del Sacro Cuore. All procedures experimental procedures had been previously approved by the Catholic University Ethical Committee for Animal research and by the Italian Ministry of Health.

Chemicals: Most of the Chemicals were obtained from Sigma-Aldrich (Milan, Italy). Sorafenib (Nexavar, BAY 43-9006) was kindly provided by Bayer Pharmaceuticals. AICAR was from Toronto Research Inc. (Toronto, ON, Canada); Compound C was from Merk Millipore (Vimondrone, Milan, Italy); Lipofectamine® 2000, 6-NBDG and H2-DCF-DA were obtained from Life Technologies Italy (Monza, MB, Italy). the HiPerFect® transfection reagent was from QIAGEN, Hilden Germany).

Antibodies and cell lines. Antibodies used for Western blotting were as follows: rabbit anti-phosho p44-42 MAP Kinase Thr202/Tyr204, rabbit anti phospho-AMPK-α Thr 172 , rabbit anti AMPK-α , rabbit anti phospho S6 (Ser 235/236) , rabbit anti LC3B and mouse monoclonal anti alpha-tubulin antibody were from Cell Signaling Technology (Danvers, MA, USA);. Anti p62/SQSTM1 mouse monoclonal antibody was from Abnova (Taipei, Taiwan). Anti myc mouse monoclonal antibody (clone 9E10) was from Santa Cruz Biotechnology Inc (Heidelberg, Germany).

LCSC-2 and 293T-POLG1(D890N) cells are described in the main Methods section.

Ras/E1A transformed, p53 -/- MEFs (clone A9) were a kind gift of Dr. Scott Lowe (CSH Laboratory, Cold Spring Harbor, NY) [1]. The other cell lines (B16F10, HEK 293-T), were obtained from ATCC/LGC Standards (Sesto San Giovanni, MI, Italy) . All cell lines, including the commercial ones, were stock-frozen upon arrival and used within few passages after resuscitation for experimental procedures.

B16F10 and E1ARas A9 (p53-/-) were maintained in Dulbecco's modified Eagle medium (DMEM 4.5 g/L d-glucose) medium supplemented with 10% FBS and 1% antibiotics. HEK 293-T (Human Embryo Kidney Cells), were maintained in Dulbecco's modified Eagle medium (DMEM 4.5 g/L d-glucose) medium supplemented with 8% FBS and 1% antibiotics.

Cells were cultured in tissue culture flasks in a humidified incubator at 37°C in an atmosphere of 95% air and 5% carbon dioxide. Cells were passaged twice a week using 0.05% trypsin/EDTA.

siRNA. A pool of 19-21 nt siRNAs specific for rat AMPKα1 (sc-270142) and a suitable scrambled control (siRNA-A, sc 37007) were obtained from Santa Cruz Biotechnology Inc

Cell transfections. For **cDNA** transfection, LCSC-2 cells were seeded at 10^5/well in 35mm glass bottom dishes (μ-Dish, cat. # 80136, IbiDi, Martinsried, Germany). After 16 hours each well was transfected with 3 μg of plasmid DNA (ptfLC3) using Lipofectamine®2000, according to manufacturer's recommendations. Cell were processed 48 hours after transfection. For **siRNA** transfections, cells were seeded at 10^5/well in 6 well clusters and transfected after 16 hours with 16.6 nM (500ng) siRNA/well using the HiPerFect® transfection reagent (QIAGEN, Hilden Germany). SiRNA transfection was repeated twice after 24 and 48 hours, and cells processed after 48 more hours.

Confocal analysis of autophagic flux in LCSC-2 cells.

The mRFP-GFP tandem fluorescent-tagged LC3 probe marks autophagosomes as yellow puncta whereas autophagolysosomes mark red because of green fluorescence quenching by the acidic lysosomal content. The ratio between yellow and red puncta reports therefore on the dynamics of autophagy flux within a cell, with an high red/yellow ratio indicating accelerated autophagy flow (see ref 24 main text). 48 hours after cell transfection and 6 hours after cell exposure to SFB Images were obtained by using an inverted confocal microscope (DMIRE2, Leica Microsystems, Germany) fitted with a 63X oil immersion objective (NA 1.4) and LCS 2.61 acquisition Software (Leica Microsystems, Germany). mRFP-GFP-LC3 was excited by an argon-ion laser (excitation wavelength: 488nm; emission ranges: 500-540 and 640-740 nm). Z-stacks were acquired every 500 nm, to allow a count of the puncta over the whole cell volume. Image analysis was performed with IMAGEJ 1.41 (NIH). "Puncta" in red and green images were identified as spots of maximum fluorescence intensity using FIND MAXIMA plugin (IMAGE-J). Upon image overlay, yellow (Red+Green) and red spots per individual cell volume were manually counted and ratio calculated.

ROS measurement. Cell were seeded at 105/well in 12 well clusters and incubated in complete or serum free medium for 16 hours. SFB was added in serum-free medium at different concentrations, for 6 hours; during the last hour H2-DCF-DA was added at 1 μg/ml. Cell were then tripsynized and resuspended in PBS, and cell fluorescence rapidly analysed by flow-cytometry (Coulter Epics® XML, ex. 480 nm, em. 530, FL-1).

6-NBDG uptake assay. LCSC-2 cells were seeded at 10^5 cells/well in 12 well cluster in medium containing 2% FCS. 16 hours later SFB was added and cells were further cultivated for 48 hours. Cells were then washed with PBS and incubated for 10-15 minutes in 300 μL of glucose free-serum free DMEM containing 100 μM of the fluorescent non hydrolyzable glucose analog 6-NBDG (6-(*N*-(7-Nitrobenz-2-oxa-1,3-diazol-4-yl)amino)-6-Deoxyglucose; Life Technologies). After 3 washes with cold PBS cells were trypsinised and immediately analysed by flow cytometry (ex. 480 nm, em. 530, FL-1).

Glucose consumption and release of L-lactate were determined by an hand-held glucometer (Accu-Check Aviva, Roche Diagnostics S.p.a., Monza, Italy) and a colorimetric kit (ScienCell Research Laboratories, Carlsbad, CA, US), respectively, in 48 hours celll supernatants containing 2% FCS. Values were normalized for number of cells in each condition (area under the 0-48h proliferation curve, N_0=100.000 cells).

Immuno Blotting. For western blot analysis cells were lysed in ice-cold lysis buffer (NaCl 150 mM, Tris-Hcl 50 mM pH 8, 2mM EDTA) containing 1% v/v Triton X-100, 0.1 % v/v SDS, 1:1.000 Protease Inhibitor cocktail (Sigma), 1 mM Sodium Orthovanadate, 1 mM NaF. After 15 minutes on ice with occasional vortexing cell lysate were cleared at 14.000 rpm, 4 °C to remove debris and unlysed cells, and supernatant quantified for protein content (DC Protein Assay, BIORAD, Segrate, Milan, Italy), resuspended in 6X Laemmli buffer, boiled and subdued SDS-PAGE Electrophoresis. After protein electrotransfer onto nitrocellulose, filters were blocked for 2 hours in TBS-T/5% w/v

skimmed dry milk, in incubated O/N with the appropriate antisera. Immunocomplexes were detected by HRP-conjugated secondary antibodies (Biorad) followed by ECL and autoradiography (GE Healthcare, Milan Italy); bands of interest were quantified by the Image J software, values were background-subtracted and normalized for the average intensity across the samples within the same filter.

RNA isolation. The total RNA was isolated from LCSC2, 24h after treatment, using the RNeasy mini kit (QIAGEN), as described elsewhere [2]. RNA was quantified using a UV spectrophotometer and RNA quality and integrity were assessed by the Agilent 2100 Bioanalyzer (Agilent, Santa Clara, CA, USA). The resulting total RNA was then used to create the biotin-labeled library to be hybridized on GeneChip Rat Gene expression 1.0 st Array (Affymetrix, Santa Clara, CA, USA), according to the recommended experimental protocols provided by the suppliers.

Microarray data analysis. The CEL files resulting from the hybridization were analyzed using the Partek® Genomic Suite™ (Partek GS). Gene-level calculation was performed by Robust Multichip Average and normalization by quantile sketch [3]. A data table (rma), together with the relative cel files and relevant information about the experiment, is available at http:// www.ncbi.nlm.nih.gov/geo/ under accession #GSE 43053. In order to identify differentially expressed genes a multiple test adjustment was applied, FDR ≤ 0.005 was set. The resulted gene list was then annotated according to the Gene Ontology (GO) database (www.geneontology.org). This allowed to assigning a category to each gene in the list, according to three defined "ontologies" (i.e. terms representing gene product properties): cellular component, biological process and molecular function. The expression of selected genes was quantified in real time PCR (qPCR) to obtain an independent validation of microarray data. Real time PCR was carried out as previously described elsewhere [2].

RT-PCR. The $2{-}\Delta\Delta Ct$ method [4] was applied to calculate fold differences (fold change, FC) in gene expression, using the gene Actin-beta (B-ACT) as the housekeeping reference for data normalization. PCR products were subjected to melting curve analysis to rule out the synthesis of unspecific products.

Statistics.

Two-tailed Student *t*-test and one- or two-way Analysis of Variance (ANOVA) with Tukey'a HSD post-hoc analysis for multiple comparisons were used where appropriate (http://vassarstats.net/). The threshold of difference significance was set at $p<0.05$.

In analyzing Sorafenib interaction with a second treatment (2DG, glucose deprivation, siAMPK etc.), the expected effect for simple additivity was calculated based on the Bliss independence model [5], modified to include a null reference (baseline cell death in untreated samples, normally ranging between 5 and 20%).

To do so, we assumed that a fixed fraction *Fb* of “spontaneous” cell death occurred independently of other stimuli in all experimental conditions. Thus, according to the independence model, the measured fractional effect of drug 1 (m_1) can be cleared from the background fraction according to the formula

$$m_1 = (F_1 + F_b) - F_1 F_b \qquad (1)$$

where F_1 is the effect fraction entirely attributable to drug 1 .

From eq (1) we can derive the expression for F_1

$$F_1 = \frac{(m_1 - F_b)}{1 - F_b} \qquad (2)$$

The same calculation can be applied to drug 2, $F_2 = \frac{(m_2 - F_b)}{1 - F_b}$ and to the combination of both drugs $\bar{F}_{1,2} = \frac{(m_{12} - F_b)}{1 - F_b}$

F_1 and F_2 are then used to calculate the predicted additivity, based on the formula

$$F_{1,2} = (F_1 + F_2) - F_1 F_2$$

where $F_{1,2}$ is the fractional additive effect and F_1 and F_2 are the fractional effects of the two drugs added separately[5]. Drug or treatment combinations were considered synergistic when their fractional effect $\bar{F}_{1,2}$, cleared of F_b as shown above, was higher than $F_{1,2}$.

Supplementary References

Reference List

1. Pani,G. *et al.* Deregulated manganese superoxide dismutase expression and resistance to oxidative injury in p53-deficient cells. *Cancer Res.* **60**, 4654-4660 (2000).

2. Bernardini,C. *et al.* Transcritpional effects of S100B on neuroblastoma cells: perturbation of cholesterol homeostasis and interference on the cell cycle. *Gene Expr.* **14**, 345-359 (2010).

3. Bolstad,B.M., Collin,F., Simpson,K.M., Irizarry,R.A., & Speed,T.P. Experimental design and low-level analysis of microarray data. *Int. Rev. Neurobiol.* **60**, 25-58 (2004).

4. Livak,K.J. & Schmittgen,T.D. Analysis of relative gene expression data using real-time quantitative PCR and the 2(-Delta Delta C(T)) Method. *Methods* **25**, 402-408 (2001).

5. BLISS,C.I. The calculation of microbial assays. *Bacteriol. Rev.* **20**, 243-258 (1956).

Supplementary figure legends.

Supplementary Figure S1. **Microarray analysis of gene expression in LCSC-2 cells exposed for 24 to SFB**. The general experimental design and the most significant GO (Gene Ontology) classes revealed by gene enrichment analysis (alongside with the corresponding p-value) are depicted. Differentially expressed genes belonging to the “monosaccharide metabolic process” and

“regulation of cell proliferation” GO classes are listed in detail in arrowed boxes. Data analysis was performed by the DAVID (**D**atabase for **A**nnotation, **V**isualization and **I**ntegrated **D**iscovery) on-line bioinformatic tool.

Supplementary Figure S2 Sorafenib toxicity as a function of extracellular glucose concentration.

LCSC-2 cells were challenged with SFB, the MEK inhibitor PD98059, or vehicle alone (1:2000 v/v Dmso) in the presence of 10 mM, 1mM or no glucose. Cell death was assessed by PI exclusion assay, after 24 hours of incubation in serum-free medium.. Compared to 10mM glucose, complete glucose deprivation enhances SFB toxicity, while low (1mM) glucose has the opposite effect. The dashed line indicates the predicted value of additivity between glucose and SFB; white circles indicate treatment-specific cell death calculated as described in Supplementary methods. No effect of Dmso (SFB vehicle) or the MEK inihibitor were observed at the indicated concentrations. Bars are mean±SD of duplicate samples . ** $p<0.01$ versus glucose 10 mM; significance for the (SFB x Glucose) interaction is indicated. Statistics by two-way ANOVA and Tukey’s *post hoc* test. Representative of several independent experiments.

Supplementary Figure S3. **Synergystic effect of Sorafenib and 2-deoxyglucose on HEK 293-T (human embryonic kidney, large-T transformed) and E1ARas A9 (Murine embyonic fibroblasts, p53-/-) cell lines.**

a and **b.** Percentage of dead cells under the indicated treatments was determined by flow cytometry, after 24 hours incubation in serum-free medium. Bars are mean SD of duplicate samples. Panels representative of two or more independent experiments. SFB=5 μM; 2DG=20mM.

For the meaning of the dashed line and the white circles see legend to S2

** $=p<0.01$ and *= $p<0.05$ compared to the corresponding Dmso group.

Differences between groups and interactive effect were calculated by two-way ANOVA and Tukey's post hoc test.

Supplementary Figure S4. **Effect of SFB and 2DG on the AMPK/mTOR cascade in B16F10 cells.**

Western blot analysis of protein lysates from B16F10 mouse melanoma cells stimulated for 4 hours with the indicated combinations of SFB and 2DG. Phosphorylation of AMP (Ser 172) and of the mTOR effector S6 (Ser 235/236) were monitored by phospho-specific antibodies as in figure 5A. Total AMPK amount throughout the samples is also displayed. Specular changes of pAMPK and pS6, consistent with activated AMPK inhibiting the mTOR cascade, and the combinatorial effect of SFB and 2DG on these changes are noteworthy. Picture representative of at least two independent experiments.

Supplementary Figure S5.

Quantitation of western blot data from Figure 5. For each protein , band densitometric values were normalized for the average value of that band in the entire sample set. Bars are average±SD of 3-5 indipendent experiments performed under identical conditions. Cell lines, bands of interest and treatment combinations are indicated and correspond to those displayed in Fig. 5.

Supplementary Fig. S6

MTT reduction assay illustrating cell protective effects of Ebselen on LCSC-2 cells exposed to SFB, but not the SFB+2DG combination. Cell were incubated for 24 hours in the presence of the indicated drug combinations, in serum-free medium. Bars are mean±SD of background-substracted absorbances from quadruplicate wells. Statistics by rwo-way ANOVA. Ebselen was protective (significant row effect) against SFB-induced growth inhibition, but not against the SFB+2DG combination. Picture representative of 2 independent experiments.

Supplementary Figure S1

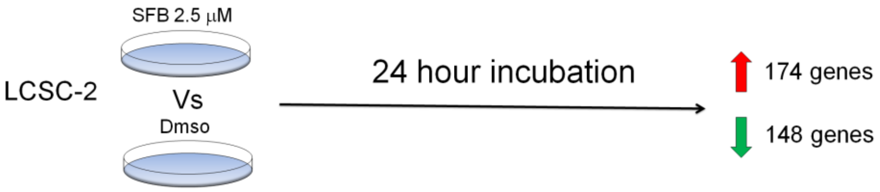

Cathegory: Biological Process

Term	Count	PValue
GO:0043627~response to estrogen stimulus	11	1,59E-12
GO:0010033~response to organic substance	31	2,46E-12
GO:0048545~response to steroid hormone stimulus	15	3,11E-11
GO:0032355~response to estradiol stimulus	8	4,12E-11
GO:0030879~mammary gland development	8	8,73E-11
GO:0035239~tube morphogenesis	10	0,00101
GO:0060562~epithelial tube morphogenesis	8	0,00119
GO:0048729~tissue morphogenesis	12	0,00128
GO:0045662~negative regulation of myoblast differentiation	3	0,00157
GO:0051094~positive regulation of developmental process	14	0,00183
GO:0060560~developmental growth involved in morphogenesis	4	0,00207
GO:0060429~epithelium development	12	0,00228
GO:0046660~female sex differentiation	7	0,00237
GO:0045596~negative regulation of cell differentiation	11	0,00258
GO:0016197~endosome transport	5	0,00440
**GO:0005996~monosaccharide metabolic process**	**11**	**0,00462**
GO:0045056~transcytosis	3	0,00531
GO:0009408~response to heat	6	0,00539
GO:0048732~gland development	10	0,00585
**GO:0042127~regulation of cell proliferation**	**21**	**0,00631**
GO:0060688~regulation of morphogenesis of a branching structure	4	0,00679
GO:0006805~xenobiotic metabolic process	4	0,00679
GO:0009266~response to temperature stimulus	7	0,00687
GO:0045661~regulation of myoblast differentiation	3	0,00700
GO:0032092~positive regulation of protein binding	3	0,00700
GO:0035295~tube development	11	0,00771
GO:0046545~development of primary female sexual characteristics	6	0,00853
GO:0045597~positive regulation of cell differentiation	11	0,00857
GO:0043009~chordate embryonic development	14	0,00889
GO:0009410~response to xenobiotic stimulus	4	0,00947
GO:0009725~response to hormone stimulus	17	0,00967
GO:0009792~embryonic development ending in birth or egg hatching	14	0,00997

RENBP, CRYAB, UGT1A5, PRKAG2, ENO2, PFKP, CHST4, PDHA1, ACN9, FABP5, GLB1

DLC1, SAT1, BMP4, TXNIP, CDX2, TBX3, MARCKSL1, BTC, ESR1, PAWR, ZEB1, RB1, CXCL10, LIF, PGR, CTH, IRF6, BTG1, SCIN, GRPR, AREG

Supplementary Figure S2

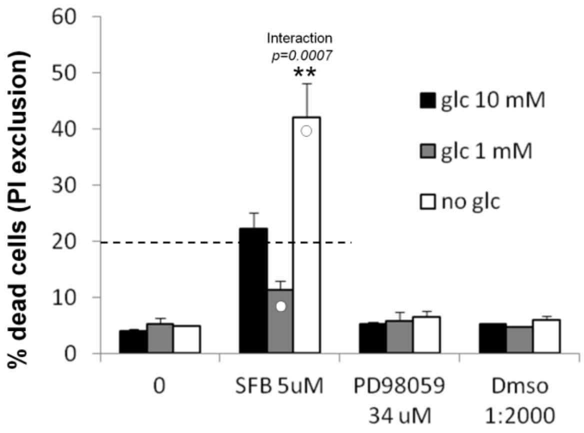

Supplementary Figure S3

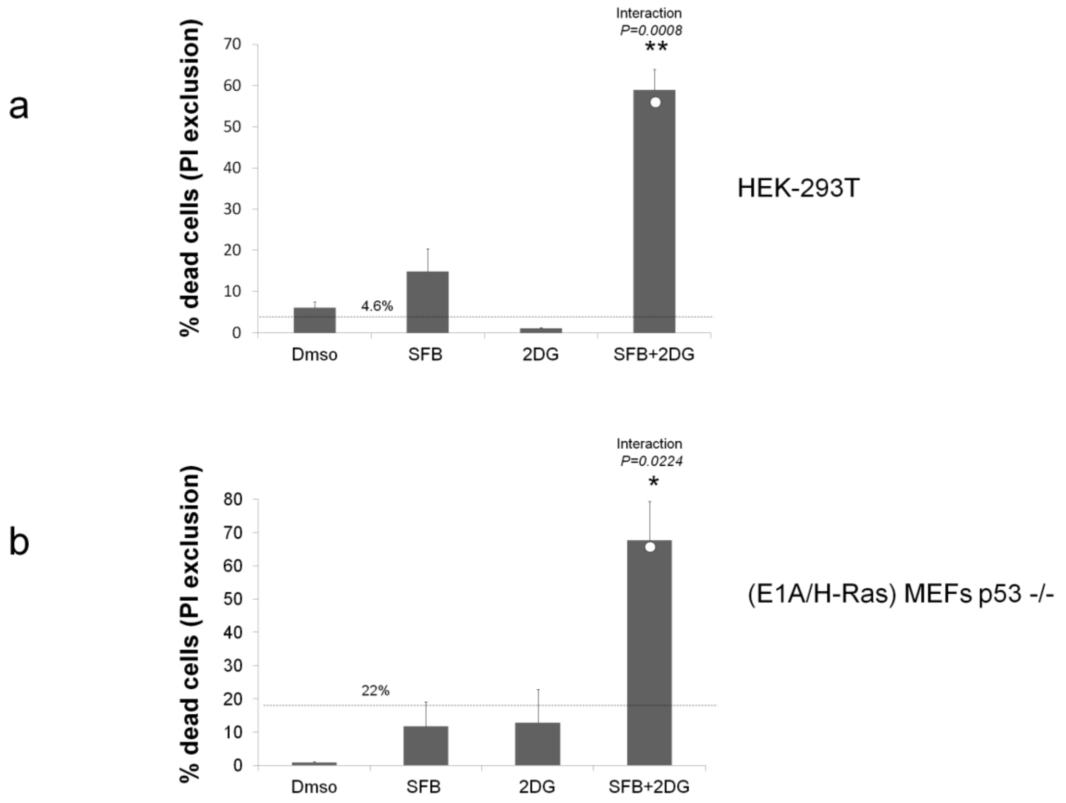

Supplementary Figure S4

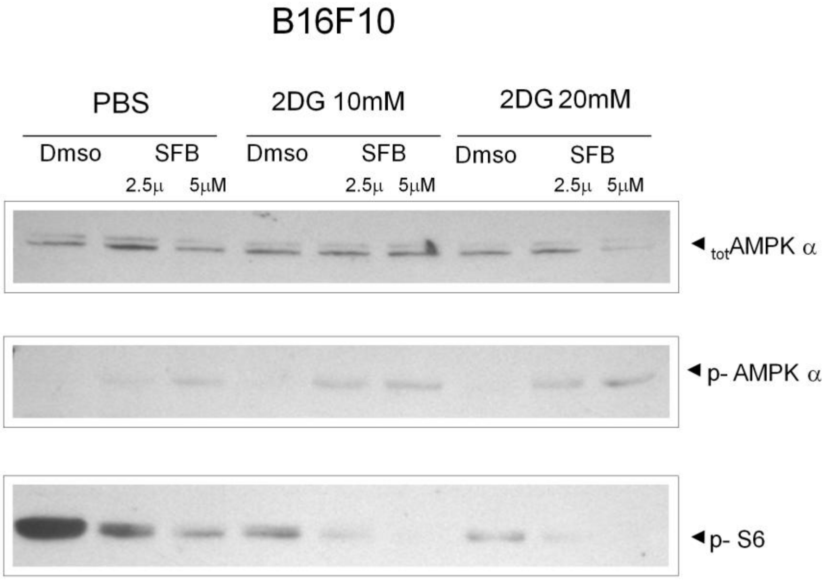

Supplementary Figure S5

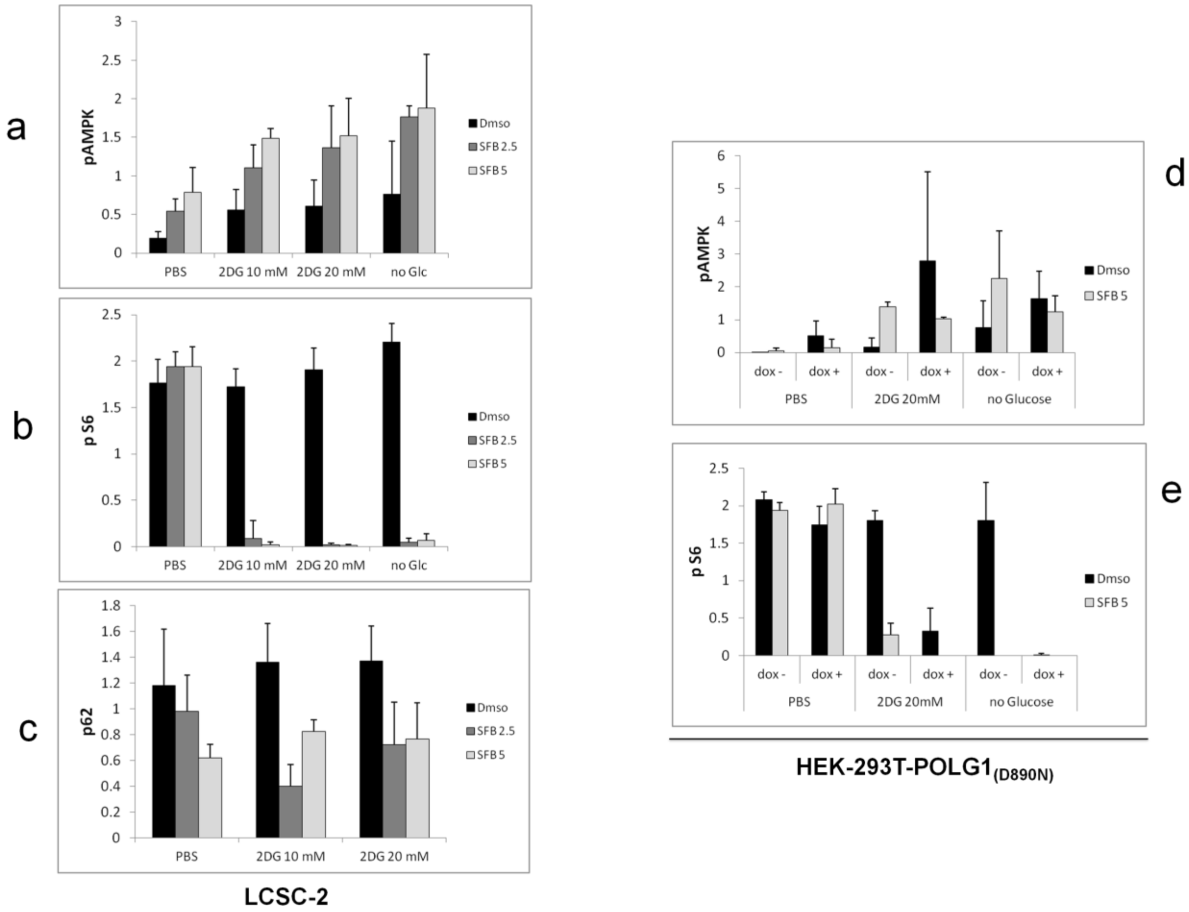

Supplementary Figure S6

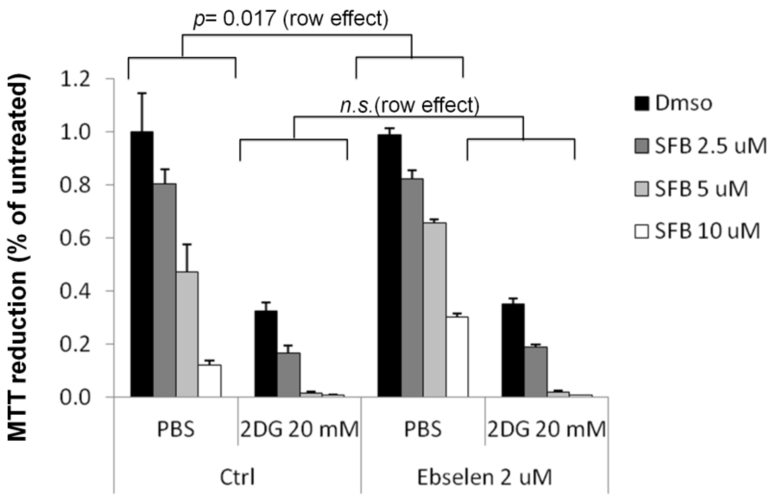

Supplement: Supplementary Information [file srep09149-s1.pdf]
